# Identification of reindeer fine-scale foraging behaviour using tri-axial accelerometer data

**DOI:** 10.1186/s40462-022-00339-0

**Published:** 2022-09-20

**Authors:** Heidi Rautiainen, Moudud Alam, Paul G. Blackwell, Anna Skarin

**Affiliations:** 1 Department of Animal Nutrition and Management, Swedish University of Agricultural Sciences, Uppsala, Sweden; 2 School of Information and Engineering, Dalarna University, Falun, Sweden; 3 School of Mathematics & Statistics, University of Sheffield, Sheffield, UK

**Keywords:** Activity recognition, Tri-axial accelerometer, Random forests, Support vector machines, Hidden Markov models, *Rangifer tarandus*

## Abstract

Animal behavioural responses to the environment ultimately affect their survival. Monitoring animal fine-scale behaviour may improve understanding of animal functional response to the environment and provide an important indicator of the welfare of both wild and domesticated species. In this study, we illustrate the application of collar-attached acceleration sensors for investigating reindeer fine-scale behaviour. Using data from 19 reindeer, we tested the supervised machine learning algorithms Random forests, Support vector machines, and hidden Markov models to classify reindeer behaviour into seven classes: grazing, browsing low from shrubs or browsing high from trees, inactivity, walking, trotting, and other behaviours. We implemented leave-one-subject-out cross-validation to assess generalizable results on new individuals. Our main results illustrated that hidden Markov models were able to classify collar-attached accelerometer data into all our pre-defined behaviours of reindeer with reasonable accuracy while Random forests and Support vector machines were biased towards dominant classes. Random forests using 5-s windows had the highest overall accuracy (85%), while hidden Markov models were able to best predict individual behaviours and handle rare behaviours such as trotting and browsing high. We conclude that hidden Markov models provide a useful tool to remotely monitor reindeer and potentially other large herbivore species behaviour. These methods will allow us to quantify fine-scale behavioural processes in relation to environmental events.

**Supplementary Information:**

The online version contains supplementary material available at 10.1186/s40462-022-00339-0.

## Introduction

Monitoring animal behaviour enables a better understanding of animal behavioural ecology in an evolutionary context e.g., inter- and intra-specific interactions such as competition and population dynamics [1]. Investigation of fine-scale animal behaviour can improve the understanding of animals’ functional response to the environment [2] and provide an important indicator of animal welfare [3]. Initial responses to stressors related to changes in animal management or environment are often behavioural and can provide the first indications of stress or impaired health of an individual [4, 5]. Tri-axial acceleration sensors have been frequently used to study fine-scale animal behaviours in both wild [6, 7] and domesticated species [8, 9]. As an example, many studies have successfully been able to classify foraging behaviour in a range of species such as harbour seals (*Phoca vitulina*), arctic ground squirrels (*Urocitellus parryii*) and roe deer (*Capreolus capreolus*) [10–12]. However, each sensor type needs validation to confirm and quantify its capacity to accurately classify specific species behaviours.

Reindeer and caribou (*Rangifer tarandus*) is a key species inhabiting the circumpolar north [13]. With the rapid and extreme climate and environmental change going on in the arctic and subarctic regions, there is a need to understand how this affects reindeer behaviour. Reindeer are ruminants of an intermediate, opportunistic feeding type [14], mainly feeding on fresh herbal plants and graminoids and to some degree browsing shrubs and trees in summer and adapted to feed mainly on ground and arboreal lichens in winter [15]. Knowledge of reindeer fine-scale grazing could for example reveal reindeer's ability to suppress the increased growth of woody taxa in the arctic [16, 17] and their ability to search for lichens [15]. Domesticated reindeer in free-roaming systems provide an excellent opportunity to validate accelerometers for *Rangifer* taxon aligning specific behaviours to the accelerometer data. Acceleration sensors have previously been used on reindeer and caribou for estimation of activity patterns [18–20], but have not yet been validated for prediction of fine-scale foraging behaviour.

The acceleration (in m/s^2^ or G-forces (g)) measured by a sensor in three dimensions (X, Y and Z) [Reviewed by: 21], may be separated into both static and dynamic acceleration [22, 23]. Animal body orientation may be registered using the static acceleration caused by gravitational force acting on the accelerometers [24, 25]. Removing the gravitational component, the dynamic acceleration is revealed. This makes it possible to identify patterns in the acceleration waveform that corresponds to an observed behaviour [26, 27].

Supervised machine learning (ML) algorithms are effective ways to classify features of animal acceleration data into pre-defined behavioural categories [28–32]. To train and validate such algorithms, movement data is collected using acceleration sensors on the animal at the same time as the animal behaviour is recorded through direct observation [33, 34], or with a camera [35]. Animal behaviour is classified into different behavioural categories and then accelerometer data is annotated with the recorded behavioural categories [36, 37]. The raw acceleration annotated with the corresponding behaviour is normally pre-processed (using running means [38, 39] or low- and high pass filters [22, 40]) to reduce noise or to separate static and dynamic acceleration. Then the data is segmented into windows, followed by extraction of characteristics of acceleration data (features), selection of features, and modelling [41]. The features and their corresponding classified behaviours are used to train the models, which learn to distinguish between the classified behaviours given the differences in the acceleration data [41]. Once a model is trained, it can be used on new data (e.g., new individuals) to quantify different behaviours performed by the animal. This enables fine-scale behavioural studies over long time periods under conditions where direct observations are difficult due to constraints such as visibility or geographic scale [20, 21, 42].

Generally, the performance of behaviour classification relies on a fixed placement and orientation of the sensors [43, 44]. This can be achieved using harnesses, halters, or glue-on tags [37, 45–47]. However, often sensors are attached to a collar around the neck of the animal [8, 48], and then it is likely that the sensor will change its position and orientation relative to the animal’s body orientation. This may cause significant errors and reduced recognition rate [49]. The variability caused by displacement of the sensors may be accounted for by using robust features derived from the raw sensor output. For example, the net acceleration computed from all three axes (the Euclidean norm of the acceleration vector) is less sensitive to changes in sensor orientation or placement [50, 51]. Alternatively, information about angles around X (roll) and Y (pitch) using a gyroscope or magnetometer can be used to correct the accelerometer sensor displacements using rotation matrices [52, 53]. Such correction has to our knowledge seldom been applied on data collected with collar-attached sensors.

We equipped reindeer with collar-attached accelerometers and registered their behaviour using video cameras to find the best model predicting reindeer foraging behaviour, such as grazing on ground lichens and browsing on shrubs and arboreal lichens in trees. The main objective of our study was to develop and validate a method for classifying foraging behaviour of reindeer using tri-axial acceleration sensors. We evaluated Random forests (RF), Support vector machines (SVM) and hidden Markov models (HMM) to find the best model to classify acceleration data into pre-defined behavioural categories.

## Methods

### Study area, animals and management

**Fig. 1:**
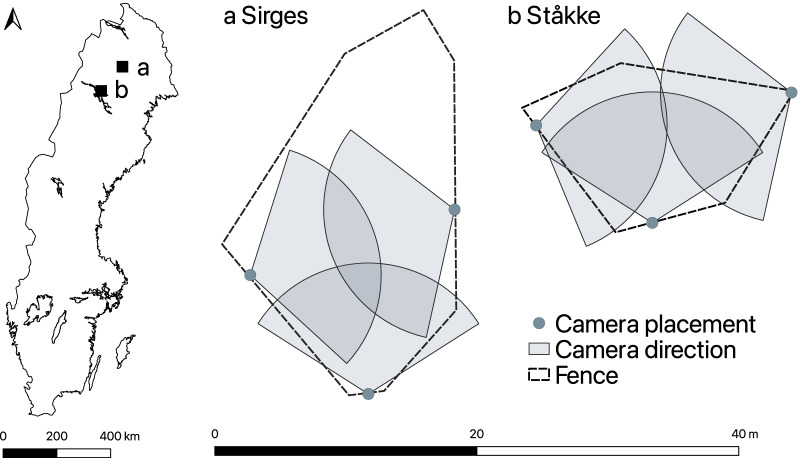
Enclosures with placement of cameras used for video recordings of ground-truth behaviour of ten and nine reindeer, respectively, fitted with acceleration sensors in (**a**) Sirges reindeer herding community and (**b**) in Ståkke reindeer herding community, both in northern Sweden

In this study, we simultaneously collected video and acceleration data from in total 19 semi-domesticated female reindeer in Ståkke and Sirges Sami reindeer herding communities in northern Sweden. Initially, ten individuals per herding community were randomly selected from two groups of 40 animals being supplementary fed in a feeding experiment conducted in each community. In Sirges, the ten reindeer (nine-month-old) were kept in a 300 m^2^ enclosure from 27 February to 2 March 2020 (Fig. 1). In Ståkke, the ten reindeer (two two-year-old and eight nine-month-old) were kept in a 150 m^2^ enclosure from 4 to 9 March 2020; one of the nine-month-old individuals was difficult to capture for collar fitting and was excluded from sensor attachment. In both enclosures, reindeer lichens (*Cladonia rangiferina* and *Cladonia arbuscula)* and commercially available pelleted reindeer feed (Renfor nära ®, Lantmännen, Sweden) were dug down under the snow to encourage natural grazing and digging behaviour. In addition, small trees covered with arboreal lichens (*Bryoria fuscescens*) were placed in the enclosures to encourage browsing behaviour.

### Video recordings

Three cameras (Axis Communications, 2025-LE Network Camera) were used and placed to cover the whole enclosure and enable video recordings of the animals from different angles. The reindeer were video recorded from 6 AM until 6 PM. In total, we generated 50 h daytime video (15 frames per second) on each individual.

### Accelerometer data

We used a three-axial accelerometer (Axy-4; 9 × 15 × 2 mm; 0.7 g) including a temperature sensor [54] positioned on the ventral-right side of the neck attached to a GPS-collar (Pellego) [55], with a total weight of 330 g. See Additional file 2: Fig. A1, illustrating the attachment and directions of the accelerometer. We choose to configure the accelerometers to a sampling rate of 10 Hz with 8-bit resolution at ± 8 g. Sampling rate of temperature was set to 0.2 Hz. At these settings, the sensor could store four months of data. Sampling rate was chosen according to Nyquist’s criterion i.e., that sampling rate should be at least twice the highest frequency component of the signal [56, 57]. Reindeer activities, like other large herbivores, were expected to involve frequencies of 5 Hz [58, 59]. Thus, sampling frequency of a minimum of 10 Hz was required to detect motions. The same computer was used for calibration and time synchronization of the accelerometer internal clock. All accelerometers were shaken in front of all cameras prior to attachment to acquire a reference for time synchronization [60]. Collars were attached to mimic the conditions surveying freely ranging reindeer, when the size of the collar needs to allow for growth of the neck, as these reindeer were still in their growth stage. Acceleration data was retrieved using Axy Manager Version 1.8.3.0 [60].

### Behavioural observations

An ethogram was created after consulting the reindeer herders about typical reindeer behaviours and two hours of behavioural observations in the enclosures (Table 1). In total, 39 h of acceleration data from 19 individuals were annotated into 17 behavioural categories. On an average two hours of annotations were performed for each individual from the first day of video recordings. Behaviours were first annotated into the main categories: browsing high, browsing low, grazing, digging, lying, standing, moving, agonistic behaviour, scratching head against tree, other and missing data. If a behaviour did not fit the listed behaviours or if an animal expressed more than one of the listed behaviours at the same time, we annotate it as “other”. The latter occurred on a few occasions when one reindeer was digging and grazing at the same time. Total number of recorded behaviours for each individual are presented in Additional file 1: Table A2. Video recordings were annotated using BORIS Version 7.9.8 [61]. Reindeer have a polycyclic activity pattern with all typical behaviours occurring in bursts both day and night [62, 63] throughout the year [64]. Thus, we expect to cover the most common behaviours occurring in night-time from the daytime recordings.

**Table 1:** Main behavioural categories of 19 video recorded reindeer attached with tri-axial accelerometers used for model training and corresponding subgroups (behavioural categories) included within each main behaviour

Behaviour	General description	Subgroup	Description
Grazing	Lower the head to the ground and foraging from the ground. Mouth close to the ground		From ground while standing still or taking one or two steps without moving head position or while walking slowly and foraging from the ground. Mouth positioned close to the ground
Browsing high	Moving lips towards a branch in a tree or a high shrub		Standing on all four legs, stretching the neck upwards, head level above shoulder height (minimum 45º head angle) or standing on the hind legs, stretching the neck upwards
Browsing low	Moving lips towards a low branch in a tree or a low shrub		Standing on all four legs, moving the head forward or downwards without mouth touching the ground
Inactivity (lying)	Belly or side on the ground with folded or extended legs and head in different positions	Resting	Folded legs with head raised from the ground facing forward or with the neck bent on the side
		Sleeping	Head close to ground (on ground or against body) in the same position
		Ruminating	Lying with legs folded and belly on the ground, head raised from the ground facing forward or with the neck bent on the side while chewing
		Grooming^a^	Lying with legs folded and belly on the ground, head moving against legs or body
Inactivity (standing)	Standing on all four legs without moving forward without chewing		
Walking	Moving forward by alternately moving the legs from one point to another		Lifting all four legs in a symmetric movement and moving forward, with mouth up from the ground (not grazing)
Trotting	Moving forward by alternately moving the legs from one point to another		Simultaneous movement of hoof paired two by two diagonally (trotting) or three-beat gait faster than the average trot (running)
Digging ^a^	Standing and repetitively scratching on ground with one front leg at least two times in a row		
Agonistic behaviour ^a^	Pushing away an individual or being pushed away by another individual		
Scratching head against tree ^a^	Repeated head movement against branches on trees without having contact with the lips on branch		
Missing data ^a^	Animal out of sight		
Other ^a^	Undefined		

^a^ Behaviours classified as “other”.

### Behaviours used in model training

Closely related behaviours with high similarity in acceleration waveforms were merged before model training i.e., grazing behaviour included grazing, grazing from a hole, and grazing while walking, and inactivity included sleeping, ruminating, standing, and resting. Running was merged with trotting due to low occurrence. To quantify reindeer foraging behaviour, a total of seven remaining behavioural categories were used for model training: grazing, browsing low, browsing high, inactivity, walking, trotting, and other behaviours. Behaviours such as walking in rough terrain were not observed in the video recordings and were therefore not included in the training and validation.

### Analyses of accelerometer data

Raw acceleration data (X, Y and Z) were first smoothed using a running mean of five seconds removing most of the static acceleration (gravitational component of acceleration) from the dynamic acceleration [e.g., 12, 23, 27]. The GPS device (175 g) acted as a counterweight to avoid unwanted collar rotations around the neck. However, this happened to some extent, and when reindeer are free-ranging the accelerometers will also be prone to unwanted rotations. From the estimated static acceleration, the angles around X (roll) and Y (pitch) were calculated [65; Table 2] to estimate accelerometer orientation. To validate our estimated angles, our filtering method and equations for pitch and roll were compared with true angles derived from a dataset collected with IMU sensors (accelerometer and gyroscope; Byström, unpublished data). To adjust for the position of the accelerometer when a collar had rotated, a rotation matrix around the X-axis was calculated to transform the sensor’s measurements into fixed measures based on the estimated angle (α) around the X-axis (Table 2). The $\ell^{2}$-norm of raw accelerometer axes was calculated to assess an orientation-independent index of acceleration magnitude [49, 66].

**Table 2:** Processing (A) performed on raw acceleration data after applying a sliding window of five seconds prior to segmentation and summary statistics (features) calculated (B) for each window (two-, three-, and five-second windows) after segmentation

A	Data processing	Term	Equation	Description
	Static acceleration	sX, sY, sZ	${\mathrm{sX}}_{\mathrm{i}}=\frac{1}{51}\sum_{\mathrm{i}-25}^{\mathrm{i}+25}{\mathrm{X}}_{\mathrm{i}}$	Gravitational component of acceleration (9.81 m/s^2^ = 1 g) caused by gravitational force acting on the accelerometers [16, 17, 35]
	Dynamic acceleration	dX, dY, dZ	${\mathrm{dX}}_{\mathrm{i}}=\left|{\mathrm{X}}_{\mathrm{i}}-{\mathrm{sX}}_{\mathrm{i}}\right|$	Dynamic acceleration measures acceleration caused by animal movements where the gravitational component is removed [e.g., 12, 23, 27]
	Roll (φ)	roll	$\mathrm{atan}2\left(\mathrm{sY},\mathrm{ sZ}\right)$	Rotation around the X-axis (roll) given in Euler angles ranging between ± π radian (equivalent to ± 180º) using 2-argument arctangent function, implemented as atan2 in R
	Pitch (θ)	pitch	$-\mathrm{atan}(\frac{\mathrm{sX}}{\sqrt{{\mathrm{sY}}^{2}+{\mathrm{sZ}}^{2}}})$	Rotation around the Y-axis (pitch) given in Euler angles ranging between ± π/2 rad (equivalent to ± 90º) using arctangent function, implemented as atan in R
	$\ell^{2}$-norm of raw accelerometer axes	Norm	$\sqrt{{\mathrm{X}}^{2}+{\mathrm{Y}}^{2}+{\mathrm{Z}}^{2}}$	Orientation-independent measure of acceleration magnitude [42, 62]
	Rotation matrix	R_x_($\varphi$)	$$\left[\begin{array}{ccc}1& 0& 0\\ 0& \mathrm{cos}\varphi & -\mathrm{sin}\varphi \\ 0& \mathrm{sin}\varphi & \mathrm{cos}\varphi \end{array}\right]$$	Rotation matrix around X-axis to adjust for rotations around the neck

B	Summary statistics	Term	Description
	Mean	mean	Mean value for each axis in each window
	Minimum	min	Minimum value for each axis in each window
	Maximum	max	Maximum value for each axis in each window
	Median	m	Median for each axis in each window
	Interquartile range	IQR	Third quantile (Q3) subtracted by the first quantile (Q1) for each axis in each window
	Standard deviation	sd	Standard deviation for each axis in each window

Acceleration data were then segmented into 2-, 3-, and 5-s windows using fixed-size non-overlapping sliding windows [67]. As a result, behaviours occurring during short timespans (< 2 s) were dropped from the data after segmentation. See Additional file 1: Table A3–A5, for final number of windows for each behaviour and individual when using 2-, 3-, and 5-s windows, respectively. Summary statistics were calculated from each segment of data resulting in 50 features. To avoid overfitting and computational load, we removed highly correlated features and used 12 features for model training and validation: m_roll, IQR_roll, mean_sX, sd_sX, mean_dX, max_dX, m_Y, IQR_Y, min_sY, max_sY, sd_dZ and mean_dZ, with m_roll being the combination of median (m, Table 2B) and Roll (roll, Table 2A) and so forth. To further decrease predictor variables to avoid overfitting, we selected the most influential variables for classification using forward feature selection for each window size implemented with the “CAST”-package [68]. Distribution of annotated data using three features (mean_sX, IQR_Y, and sd_dZ) and 2 s windows are shown in Additional file 2: Fig. A2. All data processing and analyses were performed using R version 4.0.3 [69] and RStudio version 1.3.1093 [70]. In this study, time-domain metrics were considered.

### Random forests

Random forests (RF) is a classification method that combines an ensemble of classification trees [71, 72]. Each classification tree defines decision rules to partition the dataset into subsamples with similar properties. A RF randomly selects observations and features to build multiple classification trees from a dataset. The predictions of each individual tree are averaged to give an overall classification decision [71]. Thus, the RF corrects for overfitting. In addition, RF is robust with respect to noise [71]. We initially constructed 500 trees using the “randomForest”-package [73], and used the “caret”-package [74] to tune the number of variables chosen at each iteration.

### Support vector machines

Support vector machines (SVM) is a supervised machine learning algorithm which finds optimal separating hyperplanes (decision boundaries) that separate the data points into the different classes. We implemented multiclass SVM using the “caret”-package [74] and radial kernel function using the “kernlab”-package [75]. Tuning was performed using the “caret”-package [74] to find the optimal regularization parameter C and kernels smoothing parameter gamma.

### Hidden Markov models

An hidden Markov model (HMM) is a stochastic time-series model involving an observable state-dependent process and an underlying, unobservable state process. The goal is to learn about the hidden states (in our case behaviours) by observing the state-dependent process [acceleration metrics; 26]. The hidden states are assumed to follow a first-order Markov chain, and the probability distribution of an observation at time $t$ is assumed to depend only on the state at time $t$, independently of all other observations and states [76, 77]. Thus, HMM takes into account the serial dependence between observed behaviours, unlike RF and SVM.

To fit an HMM, we need to specify the number of states and the form of the observation distributions. In our case, the number of states is the number of pre-specified behaviours, and we used state-dependent multivariate normal distributions for the observations. The transition probability matrix of the Markov chain and the parameters of the observation distributions can then be estimated by maximum likelihood, using the Forward Algorithm to evaluate the likelihood efficiently. Given a fitted HMM, the Viterbi algorithm can be used to reconstruct the most likely states (behaviours) corresponding to the observations. See for example Leos-Barajas et al. [26].

### Training and validation

To account for variability among individuals used in the training and to make generic predictions on new (unseen) individuals, we used leave-one-subject-out cross-validation for model evaluation. This ensured that an individual never occurred in the training and validation dataset at the same time for each iteration of *k*. Thus, data from one individual was always left out in each fold and was utilized as a test set. This was repeated until data from all individuals were classified. To retain the naturally unbalanced behaviours across individuals, the dataset was not balanced across individuals, and we used an unequal number of behavioural classes from each individual. Confusion matrices [${n}_{ij}$] were used to summarize model performances where $i, j$ denotes the number of observations belonging to ground-truth behaviour $i$ that were predicted by the model to be behaviour $j$ (see calculations in Additional file 1: Table A1). We computed behaviour-specific sensitivity, precision and accuracy and overall accuracy across behaviours. Cross-validation was implemented using the “CAST”-package [68] for RF and SVM.

## Results

### Model development and evaluation

Random forests was tuned for the optimal number of variables chosen at each iteration (2 s windows: mtry = 2, 3 s windows: mtry = 3, and 5 s windows: mtry = 4) and the number of trees was set to 50 (ntrees = 50, Additional file 2: Fig. A3). Future feature selection using RF to find the most important variables out of twelve resulted in eight predictor variables used for 2 s windows (mean_sX, sd_dZ, IQR_Y, IQR_roll, max_dX, min_sY, sd_sX and m_Y), nine predictor variables used 3 s windows (mean_sX, sd_dZ, IQR_Y, IQR_roll, max_dX, min_sY, sd_sX, mean_dX and m_Y), and seven predictor variables used for 5 s windows (mean_sX, sd_dZ, IQR_Y, IQR_roll, max_dX, sd_sX and min_sY). This subset of variables was later used for SVM and HMM. For RF, overall accuracy was 100% (Kappa = 1) in the training datasets for all sliding windows (2 s, 3 s and 5 s). Model overfitting was checked for RF by reducing number of trees, and the result did not differ (qualitatively) with number of trees as small as 50. Regions of tuning parameter gamma ($\gamma$) and optimal regularization parameter C (default values for C were 0.25, 0.5, 1, 2, 4) passed from training were used for SVM (2 s windows: $\gamma$ = 0.225, C = 1, 3 s windows: $\gamma$ = 0.217, C = 1, and 5 s windows: $\gamma$ = 0.308, C = 0.5). Further tuning of hyperparameters for SVM provided better overall accuracy but increased bias towards dominant classes and were not able to classify undersampled behaviours. Similarly, bias towards dominant classes increased with increased window size (Table 3). In the training dataset for SVM, overall accuracy and Kappa was 86% and 79% for 2 s windows, 89% and 83% for 3 s windows and 88% and 81% for 5 s windows, respectively. Training set accuracy and Kappa for HMM was 82% and 73% for 2 s windows, 82% and 73% for 3 s windows and 80% and 68% for 5 s windows, respectively.

**Table 3:** Performance statistics (%) of Random forests (RF), Support vector machines (SVM) and Hidden-Markov models (HMM) using time-domain features in 2-, 3- and 5-s windows (2 s, 3 s and 5 s)

		Grazing			Browsing high			Browsing low			Inactivity			Walking			Trotting			Other				
	Window size	Se	Pr	Ac	Se	Pr	Ac	Se	Pr	Ac	Se	Pr	Ac	Se	Pr	Ac	Se	Pr	Ac	Se	Pr	Ac	K	Overall accuracy
RF	2 s	**89**	**86**	**93**	25	56	62	68	64	79	**94**	92	92	45	53	72	**57**	**61**	**78**	18	51	59	72	82
	3 s	**89**	**86**	**93**	**28**	**57**	**64**	73	70	82	**94**	92	93	**49**	**57**	73	53	58	76	**21**	**61**	**60**	75	84
	5 s	86	85	92	25	49	62	**77**	**74**	**84**	**94**	**93**	**93**	49	52	**74**	34	34	67	19	50	59	**76**	**85**
SVM	2 s	89	84	93	**14**	**50**	**57**	66	65	79	**94**	91	92	47	56	73	**33**	55	**66**	**21**	39	**60**	72	82
	3 s	**89**	**86**	**93**	9	36	54	72	68	81	**94**	**92**	**93**	**48**	**59**	**73**	22	56	61	19	43	59	74	83
	5 s	86	85	92	0	0	50	**76**	**70**	**83**	**94**	**92**	**93**	43	58	71	0	**67**	52	19	**56**	59	**75**	**84**
*HMM*	*2 s*	***85***	***88***	***91***	***79***	***29***	***89***	*53*	*80*	*75*	***95***	***90***	***92***	***75***	***51***	***86***	***78***	***53***	***89***	***40***	***44***	***69***	***72***	***82***
	3 s	**85**	**88**	**91**	68	26	83	**54**	**80**	**75**	**95**	90	92	69	39	82	79	40	89	36	40	67	**72**	**82**
	5 s	78	86	88	24	20	62	54	74	74	**95**	88	90	65	38	81	24	20	62	29	25	63	66	78

Behaviour-specific metrics are given as sensitivity (Se), precision (Pr), accuracy (Ac), and overall model performance are presented as overall accuracy and Cohen’s kappa (K)

Behaviours other than grazing, browsing high, browsing low, inactivity, walking, and trotting are included as “other” in model training

Highest behaviour-specific metrics for each model are presented in bold and the overall best performing model is highlighted in italic

### Model performance

Grazing (accuracy ≥ 88%) and inactivity (accuracy ≥ 90%) were easily identified by all three models. However, for browsing low, the models did not discriminate the behaviours to the same extent (Table 3). Most confusion was found between browsing low and browsing high for all models. For the three models applied, the highest overall accuracy (85%) was found for RF using 5 s windows (Table 3). Increasing window size (from 2 to 5 s window segmentation) improved overall accuracy of RF and SVM whereas overall accuracy of HMM increased with smaller window size. The best performing HMM (using 2 s windows) had better performance across all individual behaviours and was able to classify browsing high (Table 3). Similarly, HMM had better performance of trotting compared to the best performing RF and SVM (Table 3). Confusion matrices and F1-scores are provided in Additional file 1 (RF: Table A6, SVM: Table A7, HMM: Table A8, F1-scores: Table A9).

## Discussion

We illustrate application of collar-attached acceleration sensors to quantify reindeer fine-scale behaviour. Using data from 19 reindeer, we tested the supervised machine learning algorithms RF, SVM, and HMM to find the best model classifying reindeer behaviour. Overall, HMM performed best in predicting individual and rare behaviours, while RF and SVM were biased towards dominant classes and less able to handle rare behaviours such as trotting and browsing high.

Predicting grazing, accuracy varied between 88% (HMM) and 93% (RF and SVM). Our results were similar to Alvarenga et al. [78] and Barwick et al. [79] classifying five sheep behaviours using halter and collar-attached accelerometers, respectively. Other studies have reported prediction accuracy and sensitivity for feeding behaviours from 77% to 96% and 75% to 100%, respectively [29, 80–82]. In these studies, sheep and cow behaviour were classified and the predictive performance tended to increase with a lower number of behavioural classes included in the models [29, 80–82]. Similarly, Turner et al. [83] found a reduction in overall accuracy when more behavioural classes of sheep behaviour were included in the models using RF, SVM and Deep learning techniques. For example, RF performed best using three behavioural classes with an overall accuracy of 83%, whereas overall accuracy dropped to a maximum of 72.4% for a RF model when using nine classes. Support vector machines achieved 77% overall accuracy using three behavioural classes but dropped to 58% when using nine behavioural classes [83]. In our models, seven behavioural classes were used of which feeding was separated into three subgroups (grazing and browsing high or low). Our results also indicated that when behaviour was classified as browsing high, this was generally correct, but that actual browsing high behaviour was often classified as browsing low (see confusion matrices in Additional file 1, Table A6–A8). This most likely depended on the two behaviours only being separated by the change in angle of the head. The accelerometer was attached to the neck, and the change of the neck angle when browsing high or low might not have been large enough to separate the acceleration pattern for the two behaviours. Thus, there was not a clear difference in acceleration between the two behaviours and the sensitivity for browsing high was lower than for other behaviours only reaching 79% (HMM, 2 s windows), 28% (RF, 3 s windows) and 14% (SVM, 2 s windows). In our dataset, browsing high was also a relatively rare behaviour only expressed by a few individuals. More annotations on rare behaviours could further have improved our classification accuracy, at least for those with distinct feature characteristics. For example, by visualizing the distribution using three statistical features (Additional file 2: Fig. A2) for our seven behavioural classes, it seems like browsing high has a distinct cluster. Nevertheless, RF and SVM failed to predict browsing high.

Annotating behavioural data to the accelerometer data is time-consuming, why a common challenge is to produce a dataset with a sufficient number of observations for each behaviour across individuals. Datasets with too few ground-truth observations are prone to overfitting. In addition, without enough individuals, it may not be possible to generate generalizable results on new individuals. To overcome this problem overlapping windows may be used when segmenting the data. The common data are shared across successive time windows, usually with 50% overlap between the two windows [84]. Using 50% overlap compared to no overlap may increase classification performance significantly [85]. Bersch et al. [67] compared classification performance in human activity recognition data using 0%, 25%, 50%, 75%, and 90% overlap and found a higher accuracy with increased overlap compared to no overlap. Riaboff et al. [86] found that the best prediction performance of cow behaviour was achieved when using 90% overlap. However, there is a risk of information leakage resulting in over-optimistic results when data is shared across adjacent windows [87]. With our large dataset (90,052 labelled samples for one-second windows for seven behavioural categories) it was not necessary with overlapping windows for segmentation. Information leakage could therefore be avoided in our model training.

It is important that an animal’s movement pattern is observed over an optimal time window to be able to identify different behaviours. Hence, window size may have a significant impact on the prediction results [67]. In our evaluation of window size on model performance, we found that RF and SVM had slightly higher performance accuracies at longer window sizes, while HMM had higher performance at shorter window sizes (Table 3). Hidden Markov models consider the serial dependence between behaviours and will most likely gain more information from using shorter time windows. Thus, optimal window size depends on the model selected to classify behaviour.

We had one collar rotating clearly around the neck of the animal. If collar rotations are not accounted for this may result in significant errors when using collar-attached acceleration sensors [49]. One way of dealing with collar rotations is to use orientation-independent features such as the total magnitude of all axes [49]. There may be a risk of decreased recognition rates due to loss of dimensionality when using orientation-independent features [43], but Kamminga et al. [49] and Barker et al. [50] used orientation-independent features and found that feeding behaviour of goat and cow was predicted with an accuracy of 83% and 86%, respectively. Alternatively, rotation matrices can be used for correcting sensor rotations [52]. To reduce the effect of collar rotations, we transformed the data along the X-axis using rotation matrices enabling better discrimination between the behaviours. Thus, total magnitude of axes were removed to avoid over-fitting. Using more individuals with rotating collars and combining magnetometers and/or gyroscopes to provide the true angles, would provide further insight into the impact of using rotation matrices on model performance. Increasing the number of sensor outputs, however, shortens battery life and capacity of the sensor.

Cross-validation is necessary to evaluate the reliability of a model [88, 89]. In many studies, cross-validation is performed using random K-fold cross-validation [e.g., 29, 35], or leave-one-out cross-validation [90] when data is randomly split across individuals into training and validation data. Using these methods, it is likely that observations from all individuals are present in each fold and the model is both trained and validated on all individuals. Random K-fold cross-validation may be suitable for models that will be used to monitor the same group of individuals again. However, this tends to show over-optimistic results and may not assess a generic performance on new individuals [89]. Other studies have used a single random split of training and validation data [59, 91]. Model performance in these studies with experimental settings when animals are fenced is high (overall accuracy ranging from 95 to 98%), but may not be generalizable and good enough to be used on data from new individuals.

It may be challenging to label behaviours on enough individuals to capture the individual variation in a population, especially in wild and free-ranging species. In our study, we strived for a model that would be applicable when reindeer are free-ranging and able to predict behaviour of new individuals. Therefore, leave-one-ID-out cross-validation was used. In other words, we made sure that each fold of observations from one individual was not present in both training and validation data. Leave-one-ID-out cross-validation may increase the variance of the results and significantly reduce prediction accuracy by 40 percent due to the individual variation [89, 92]. However, it will evaluate the generic performance of the models as it includes data from unseen objects, compared to traditional K-fold cross-validation [89, 93]. To our knowledge, this is rarely implemented for classification models on animal-borne accelerometer sensors but has been used for behaviour classification of e.g., meerkat (*Suricata suricatta*), sheep and cattle [80, 94, 95]. A reason for this could be the time-consuming work of compiling a large enough dataset to perform leave-on-ID-out cross-validation as in Riaboff et al. [96].

Many machine learning algorithms are sensitive to unbalanced classes decreasing overall performance [97–99]. Therefore, stratified cross-validation to handle unbalanced datasets is sometimes used [35, 100] and is recommended by Riaboff et al. [101]. In our data, behaviours were unbalanced because some activities were not equally frequently performed across individuals. However, to retain the naturally unbalanced frequencies, behaviours were not stratified. Hidden Markov models had lower overall performance compared to RF and SVM but were able to better predict rare classes and thus under-sampled behaviours (browsing high and trotting). Compared to Smith et al. [94] implementing leave-one-subject out cross-validation using SVM for activity classification of six cow behaviours, our F-scores were higher (Additional file 1, Table A9). Other techniques during data collection could be considered, such as active learning to deal with naturally unbalanced datasets [102]. It is also possible to use a refined systematic approach during the labelling process to obtain more balanced datasets, by collecting just enough observations for each class to avoid under- and oversampling.

Our methods enabled generation of detailed classification of activity data from collar-attached acceleration sensors. Being able to document reindeer fine-scale foraging patterns have a wide range of applications. For example, in management of reindeer information on how reindeer behaviour is affected by management actions, extreme weather events, human presence, changes in habitat structure and land fragmentation are essential. As an example, supplementary feeding has become more common due to competing land use and climate change [103, 104]. Supplementary feeding might be beneficial in the short term but might risk the reindeer’s future ability to search for natural fodder such as ground lichens under the snow, especially under extreme conditions. Warm and wet weather in winter increase icing on the ground and in the snow, restricting access to ground lichens [105]. Such conditions may try reindeer foraging skills searching, finding, and digging for lichens under the snow. In addition, from a climate change perspective with increasing shrubification of the arctic tundra, our method could be vital to quantify reindeer foraging intensity on shrubs and trees and its ability to suppress this vegetative greening [17, 106].

In conclusion, classification of remote fine-scale foraging behaviours from accelerometer data provides means to answer a wide range of questions related to animal behaviour, physiology, and ecology. Our results demonstrate that behaviours can be distinguished by isolated sequences of accelerometer data applying time domain features using a sampling frequency of 10 Hz. Hidden Markov models was able to best predict behaviours based on naturally unbalanced data and thus provide a useful tool to remotely monitor reindeer behaviour and to quantify how foraging behaviour of reindeer is affected by winter feeding.

## Supplementary Information

**Additional file 1**. Contains supplementary tables including equations for performance statistics, number of recorded behaviours for each individual, confusion matrices, and F1-scores.

**Additional file 2**. Contains supplementary figures including illustrations of sensor attachment, data distribution using three statistical features, and out-of-bag error of random forests.

## Data Availability

Data are available from the Dryad Digital Repository: https://doi.org/10.5061/dryad.8sf7m0cs7.
