# Supplementary material for: Identification of reindeer fine-scale foraging behaviour using tri-axial accelerometer data
Source: Mov Ecol. 2022 Sep 20;10:40. doi: 10.1186/s40462-022-00339-0 (PMC9490970; doi:10.1186/s40462-022-00339-0)
Supplement: Supplementary file 1 — Additional file 1. Contains supplementary tables including equations for performance statistics, number of recorded behaviours for each individual, confusion matrices, and F1-scores. [file 40462_2022_339_MOESM1_ESM.pdf]

Additional file 1

Table A1. Confusion matrices with seven behavioural classes (b = 7) were used to summarize model performances. The elements n_{ij} in a confusion matrix denotes how many observations of the ground-truth (reference) behaviour i that has been classified as behaviour j. We computed behaviour-specific sensitivity, specificity and accuracy and overall accuracy across the behaviour.

**Performance statistics**	**Expression[1]**	**Equation[2]**	**Description**
Sensitivity (Se)	$\frac{\mathrm{TP}}{\mathrm{TP} + \mathrm{FN}}$	$\mathrm{Se}_j = \frac{n_{jj}}{n_{j\cdot}}$	How often behaviour *j* is correctly identified by the model
Precision (Pr)	$\frac{\mathrm{TP}}{\mathrm{TP} + \mathrm{FP}}$	$\mathrm{Pr}_j = \frac{n_{jj}}{n_{\cdot j}}$	How often the model is right when it predicts behaviour *j*
Accuracy (Ac)	$\frac{\mathrm{TP} + \mathrm{TN}}{\mathrm{TP} + \mathrm{TN} + \mathrm{FP} + \mathrm{FN}}$	$\mathrm{Ac} = \frac{\Sigma_{i=1}^{b} n_{ii}}{n_{\cdot\cdot}}$	How often the model is right, across all behaviours
Kappa (κ)	$\frac{\text{Accuracy} - \text{Random accuracy}}{1 - \text{Random accuracy}}$	$\kappa = \frac{n_{\cdot\cdot}\Sigma_{i=1}^{b} n_{ii} - \Sigma_{i=1}^{b} n_{i\cdot}.n_{\cdot i}}{n_{\cdot\cdot}{}^2 - \Sigma_{i=1}^{b} n_{i\cdot}.n_{\cdot i}}$	How overall accuracy compare to values assigned by chance
F1 score	$2 \times \frac{\text{Precision} \times \text{Sensitivity}}{\text{Precision} + \text{Sensitivity}}$		Harmonic mean of precision and sensitivity

[1] TP indicate number of positive cases correctly identified as positives (true positives), TN indicate number of negative cases correctly identified as negative (true negatives), FN indicate number of positive cases misclassified as negative (false negatives), and FP indicate number of negative cases misclassified as positive (false positives).

[2] where n_{ij} is the number of observations in row i, column j, $n_{i\cdot}$ is the total number of observations in row i, $n_{\cdot j}$ is the total number of observations incolumn j, and $n_{\cdot\cdot}$ is the total number of observations.

Table A2. Number of recorded behaviours for each individual (N = 19). Behaviour length ranges between 0.1 – 3210.8 seconds.

	Individual																		
**Behaviour**	**1**	**2**	**3**	**5**	**6**	**7**	**8**	**9**	**10**	**11**	**12**	**13**	**14**	**15**	**16**	**17**	**18**	**19**	**20**	**TOTAL**
Agnostic behaviour	14	21	20	34	2	9	6	10	11	3	25	7	2	17	9	9	7	1	3	210
Digging	00	6	28	80	2	23	48	1	9	1	0	7	0	1	2	2	21	2	7	240
Grazing	163	266	422	528	108	197	367	150	5	193	63	197	185	223	229	138	168	84	196	3880
Browsing high	7	36	23	5	10	6	65	2	120	1	5	25	0	5	19	1	0	2	0	328
Browsing low	119	101	147	160	48	90	157	85	1	12	60	93	7	92	62	25	50	130	81	1520
Missing data	34	13	27	12	22	11	7	18	57	49	31	92	93	91	67	137	113	50	76	1000
Other	41	93	44	94	7	43	90	28	60	51	82	86	37	32	48	50	78	25	37	1026
Running	0	1	2	1	0	1	1	1	50	2	2	1	0	40	1	11	0	1	1	116
Standing	70	109	320	264	38	97	155	83	110	94	143	88	159	37	149	159	168	40	86	2369
Trotting	12	2	3	5	0	4	8	1	1	79	114	12	32	194	30	125	28	7	18	675
Walking	108	120	316	134	75	93	214	71	40	165	262	116	185	63	213	97	169	95	125	2661
Grazing while walking	85	103	133	310	49	89	104	74	77	116	26	88	100	0	49	51	93	21	55	1623
Breaking branches	0	1	2	3	0	0	18	4	9	0	0	0	0	0	0	58	61	0	0	156
Grooming	8	4	11	2	0	0	4	0	0	0	0	0	0	0	0	0	0	0	0	29
Sleeping	2	4	0	2	0	0	8	0	0	0	0	0	0	0	0	0	0	0	0	16
Ruminating	7	0	12	13	0	0	2	0	0	0	0	0	0	0	0	0	0	0	0	34
Resting	4	11	14	13	0	0	16	0	0	0	0	0	0	0	0	0	0	0	0	58
TOTAL	674	891	1524	1660	361	663	1270	528	550	766	813	812	800	795	878	863	956	458	685	15947

Table A3. Number of windows for each behaviour and individual (N = 19) using two second windows. Behaviours shorter than two seconds were dropped when using two second windows.

	Individual																			
**Behaviour**	**1**	**2**	**3**	**5**	**6**	**7**	**8**	**9**	**10**	**11**	**12**	**13**	**14**	**15**	**16**	**17**	**18**	**19**	**20**	**TOTAL**
Agnostic behaviour	15	25	14	14	0	0	5	5	0	0	3	0	0	1	1	0	0	0	0	83
Breaking branches	0	1	2	4	0	0	122	6	5	0	0	0	0	0	0	154	187	0	0	481
Digging	0	3	13	34	0	6	62	0	0	0	0	0	0	0	0	0	1	1	0	120
Grooming	132	26	268	11	0	0	27	0	0	0	0	0	0	0	0	0	0	0	0	464
Grazing	463	469	764	890	280	292	869	255	190	335	78	327	109	451	554	261	213	161	230	7189
Browsing high	8	71	32	11	15	8	234	1	0	0	3	28	0	27	2	0	0	0	0	438
Browsing low	2435	746	545	211	192	308	1075	702	149	2	136	345	14	476	135	48	135	654	239	8547
Missing data	662	152	136	22	325	75	27	43	118	141	39	340	146	333	181	732	444	245	159	4320
Other	93	148	100	189	22	52	182	34	64	61	57	92	14	26	13	27	18	29	16	1237
Resting	54	2929	1661	548	0	0	2473	0	0	0	0	0	0	0	0	0	0	0	0	7665
Ruminating	3085	0	1061	1957	0	0	793	0	0	0	0	0	0	0	0	0	0	0	0	6896
Running	0	0	1	1	0	1	26	2	0	0	0	0	0	0	0	0	0	0	0	31
Sleeping	171	377	0	540	0	0	595	0	0	0	0	0	0	0	0	0	0	0	0	1683
Standing	500	381	606	1033	72	138	610	153	351	155	170	47	288	11	262	148	276	89	91	5381
Trotting	10	1	0	1	0	3	8	0	0	36	125	1	7	10	9	37	10	4	6	268
Walking	170	116	364	49	70	94	313	67	3	85	241	49	78	103	87	53	77	243	47	2309
Grazing while walking	228	50	81	176	14	19	87	17	24	21	2	9	19	11	1	10	15	6	7	797
**TOTAL**	8026	5495	5650	5695	995	1000	7513	1289	908	836	857	1238	675	1449	1245	1470	1376	1432	795	47944

Table A4. Number of windows for each behaviour and individual (N = 19) using three second windows. Behaviours shorter than three seconds were dropped when using three second windows.

	Individual																			
**Behaviour**	**1**	**2**	**3**	**5**	**6**	**7**	**8**	**9**	**10**	**11**	**12**	**13**	**14**	**15**	**16**	**17**	**18**	**19**	**20**	**TOTAL**
Agnostic behaviour	6	11	7	6	0	0	3	6	0	0	0	0	0	0	0	0	0	0	0	39
Breaking branches	0	0	1	2	0	0	77	4	1	0	0	0	0	0	0	89	101	0	0	275
Digging	0	0	6	14	0	2	28	0	0	0	0	0	0	0	0	0	0	0	0	50
Grooming	87	17	176	6	0	0	16	0	0	0	0	0	0	0	0	0	0	0	0	302
Grazing	267	285	452	525	163	161	491	272	109	187	44	186	50	253	318	151	130	105	126	4274
Browsing high	5	39	19	6	8	4	135	0	0	0	1	15	0	19	0	0	0	0	0	250
Browsing low	1599	469	337	123	112	188	683	900	89	0	80	208	8	292	73	29	83	420	147	5840
Missing data	436	95	85	12	213	45	16	52	75	83	21	206	84	201	103	456	274	153	87	2697
Other	55	77	63	111	14	29	99	34	36	33	32	47	4	12	8	12	7	20	9	702
Resting	35	1946	1105	363	0	0	1647	0	0	0	0	0	0	0	0	0	0	0	0	5096
Ruminating	2055	0	700	1302	0	0	528	0	0	0	0	0	0	0	0	0	0	0	0	4585
Running	0	0	0	0	0	1	16	0	0	0	0	0	0	0	0	0	0	0	0	17
Sleeping	114	251	0	359	0	0	398	0	0	0	0	0	0	0	0	0	0	0	0	1122
Standing	314	238	361	632	42	78	373	184	212	88	88	21	162	5	144	80	155	53	47	3277
Trotting	5	0	0	1	0	1	3	0	0	12	66	0	1	6	4	15	6	2	2	124
Walking	95	59	197	19	36	41	191	62	0	41	110	18	34	43	36	23	33	148	19	1205
Grazing while walking	136	20	44	79	3	5	53	4	12	5	1	4	4	6	0	3	7	2	2	390
**TOTAL**	5209	3507	3553	3563	594	558	4760	1522	536	449	444	705	347	837	686	858	796	903	439	30266

Table A5. Number of windows for each behaviour and individual (N = 19) using five second windows. Behaviours shorter than five seconds were dropped when using five second windows.

	Individual																			
**Behaviour**	**1**	**2**	**3**	**5**	**6**	**7**	**8**	**9**	**10**	**11**	**12**	**13**	**14**	**15**	**16**	**17**	**18**	**19**	**20**	**TOTAL**
Agnostic behaviour	1	4	2	1	0	0	1	9	0	0	0	0	0	0	0	0	0	0	0	18
Breaking branches	0	0	0	1	0	0	44	7	0	0	0	0	0	0	0	42	49	0	0	143
Digging	0	0	1	3	0	0	9	0	0	0	0	0	0	0	0	0	0	0	0	13
Grooming	51	7	100	3	0	0	9	0	0	0	0	0	0	0	0	0	0	0	0	170
Grazing	125	122	223	246	79	70	241	372	45	80	21	84	20	108	149	76	60	63	46	2230
Browsing high	2	20	6	3	3	2	68	1	0	0	0	5	0	11	0	0	0	0	0	121
Browsing low	930	259	178	56	57	94	371	902	43	0	29	96	5	152	34	14	36	242	68	3566
Missing data	250	53	49	6	119	25	7	75	32	42	9	101	36	103	53	243	136	85	40	1464
Other	29	32	29	52	5	12	45	38	16	13	8	17	0	5	1	5	1	8	2	318
Resting	21	1165	658	213	0	0	986	0	0	0	0	0	0	0	0	0	0	0	0	3043
Ruminating	1231	0	419	777	0	0	316	0	0	0	0	0	0	0	0	0	0	0	0	2743
Running	0	0	0	0	0	0	9	3	0	0	0	0	0	0	0	0	0	0	0	12
Sleeping	67	149	0	215	0	0	236	0	0	0	0	0	0	0	0	0	0	0	0	667
Standing	175	111	164	330	20	37	196	191	102	43	40	6	70	1	59	34	72	35	19	1705
Trotting	1	0	0	0	0	0	0	0	0	3	17	0	0	1	1	2	2	1	1	29
Walking	40	21	78	5	16	16	71	85	0	11	39	6	9	12	10	13	13	71	5	521
Grazing while walking	66	4	12	25	0	0	16	17	2	0	0	0	0	1	0	1	1	0	0	145
**TOTAL**	2989	1947	1919	1937	300	257	2626	1707	241	192	163	315	140	394	307	430	370	505	181	16920

Table A6. Confusion matrices to illustrate hold-out predictions using Random Forests validated using leave-one-subject-out cross validation to classify reindeer behaviours using 2, 3 and 5 non-overlapping windows. Elements in the diagonal are correctly classified by the model, and the elements outside are misclassified. Based on a data set with ground-truth data collected from 19 reindeer.

	**Random Forests using 2 second windows**							
		**Reference**						
**Prediction**		Grazing	Browsing high	Browsing low	Other	Resting	Trotting	Walking
	Grazing	7100	0	708	197	70	7	319
	Browsing high	0	110	62	7	14	2	2
	Browsing low	544	269	5810	333	1182	12	862
	Other	32	5	35	210	26	69	33
	Resting	77	51	1453	234	20243	9	27
	Trotting	3	2	7	80	1	169	16
	Walking	232	3	472	87	89	31	1050
	**Random Forests using 3 second windows**							
		Grazing	Browsing high	Browsing low	Other	Resting	Trotting	Walking
**Prediction**	Grazing	4172	0	406	84	38	3	154
	Browsing high	0	71	40	5	6	1	1
	Browsing low	334	157	4261	208	727	6	433
	Other	16	1	18	141	10	35	11
	Resting	34	18	885	142	13253	5	11
	Trotting	1	0	3	38	4	75	9
	Walking	108	4	227	48	42	16	586
	**Random Forests using 5 second windows**							
		Grazing	Browsing high	Browsing low	Other	Resting	Trotting	Walking
**Prediction**	Grazing	2040	0	225	46	20	1	65
	Browsing high	0	30	23	0	7	0	1
	Browsing low	238	81	2759	108	387	0	176
	Other	6	1	16	66	17	18	9
	Resting	25	6	426	81	7702	3	9
	Trotting	0	1	0	21	1	14	4
	Walking	66	2	117	22	24	5	257

Table A7. Confusion matrices illustrating Cross-validated predictions using Support Vector Machines with leave-one-subject-out cross validation to classify reindeer behaviours using 2, 3 and 5 non-overlapping windows. Elements in the diagonal are correctly classified by the model, and the elements outside are misclassified. Based on a data set with ground-truth data collected from 19 reindeer.

	**Support Vector Machines using 2 second windows**							
		**Reference**						
**Prediction**		Grazing	Browsing high	Browsing low	Other	Resting	Trotting	Walking
	Grazing	7118	0	737	162	77	6	341
	Browsing high	0	62	47	4	8	1	1
	Browsing low	532	305	5651	368	1062	13	786
	Other	47	13	72	244	48	147	49
	Resting	70	57	1580	229	20352	8	32
	Trotting	1	0	3	66	1	99	10
	Walking	220	3	457	75	77	25	1090

	**Support Vector Machines using 3 second window**							
		Grazing	Browsing high	Browsing low	Other	Resting	Trotting	Walking
**Prediction**	Grazing	4145	1	432	53	42	3	171
	Browsing high	0	19	26	3	4	0	1
	Browsing low	372	200	4199	281	706	32	415
	Other	22	5	40	127	23	55	23
	Resting	37	22	927	144	13258	5	17
	Trotting	1	0	0	20	1	31	2
	Walking	88	4	216	38	46	15	576

	**Support Vector Machines using 5 second window**							
		Grazing	Browsing high	Browsing low	Other	Resting	Trotting	Walking
**Prediction**	Grazing	2044	1	234	20	22	1	73
	Browsing high	0	0	2	0	0	0	0
	Browsing low	262	103	2713	164	393	23	209
	Other	4	3	8	66	18	10	9
	Resting	27	13	520	80	7704	3	4
	Trotting	0	0	0	1	0	2	0
	Walking	38	1	89	13	21	2	226

Table A8. Confusion matrices to illustrate model performance using Hidden Markov Models validated using leave-one-subject-out cross validation to classify reindeer behaviours using 2, 3 and 5 non-overlapping windows. Elements in the diagonal are correctly classified by the model, and the elements outside are misclassified. Based on a data set with ground-truth data collected from 19 reindeer.

	**Hidden Markov Models using 2 second windows**							
		**Reference**						
		Grazing	Browsing high	Browsing low	Other	Resting	Trotting	Walking
**Prediction**	Grazing	6816	0	533	113	120	3	203
	Browsing high	1	348	488	122	222	4	19
	Browsing low	445	56	4565	136	349	1	183
	Other	164	6	281	459	66	22	45
	Resting	40	16	1924	181	20502	8	60
	Trotting	23	6	43	63	13	233	61
	Walking	499	8	713	74	353	28	1738
	**Hidden Markov Models using 3 second window**							
		Grazing	Browsing high	Browsing low	Other	Resting	Trotting	Walking
**Prediction**	Grazing	3960	1	326	47	71	1	114
	Browsing high	1	170	277	64	114	5	16
	Browsing low	319	50	3136	98	222	1	114
	Other	90	9	161	237	53	8	33
	Resting	22	12	1317	110	13428	3	16
	Trotting	9	5	31	70	15	112	35
	Walking	264	4	592	40	177	11	877
	**Hidden Markov Models using 5 second window**							
		Grazing	Browsing high	Browsing low	Other	Resting	Trotting	Walking
**Prediction**	Grazing	1863	0	152	68	42	0	46
	Browsing high	0	29	70	23	22	1	3
	Browsing low	195	75	1930	152	197	0	70
	Other	144	7	254	193	92	25	44
	Resting	4	7	899	164	7734	3	7
	Trotting	1	0	3	16	6	10	14
	Walking	168	3	258	46	65	2	337

Table A9. F1 scores of Random forests (RF), Support vector machines (SVM) and Hidden-Markov models (HMM) using time-domain features in 2-, 3-, and 5-second windows (2s, 3s and 5s).

		F1 scores for each behaviour						
Model	Window size	Grazing	Browsing high	Browsing low	Resting	Trotting	Walking	Other
RF	2s	87	35	66	93	59	49	27
	3s	88	38	71	93	55	52	31
	5s	85	35	75	94	39	51	28
SVM	2s	87	22	65	93	41	51	28
	3s	87	13	70	93	32	53	26
	5s	86	NA	73	93	1	50	26
HMM	2s	86	42	64	92	63	61	42
	3s	86	38	64	93	54	55	38
	5s	82	22	62	91	22	48	27
