# Supplementary material for: Identification of reindeer fine-scale foraging behaviour using tri-axial accelerometer data
Source: Mov Ecol. 2022 Sep 20;10:40. doi: 10.1186/s40462-022-00339-0 (PMC9490970; doi:10.1186/s40462-022-00339-0)
Supplement: Supplementary file 2 — Additional file 2. Contains supplementary figures including illustrations of sensor attachment, data distribution using three statistical features, and out-of-bag error of random forests. [file 40462_2022_339_MOESM2_ESM.pdf]

Additional file 2

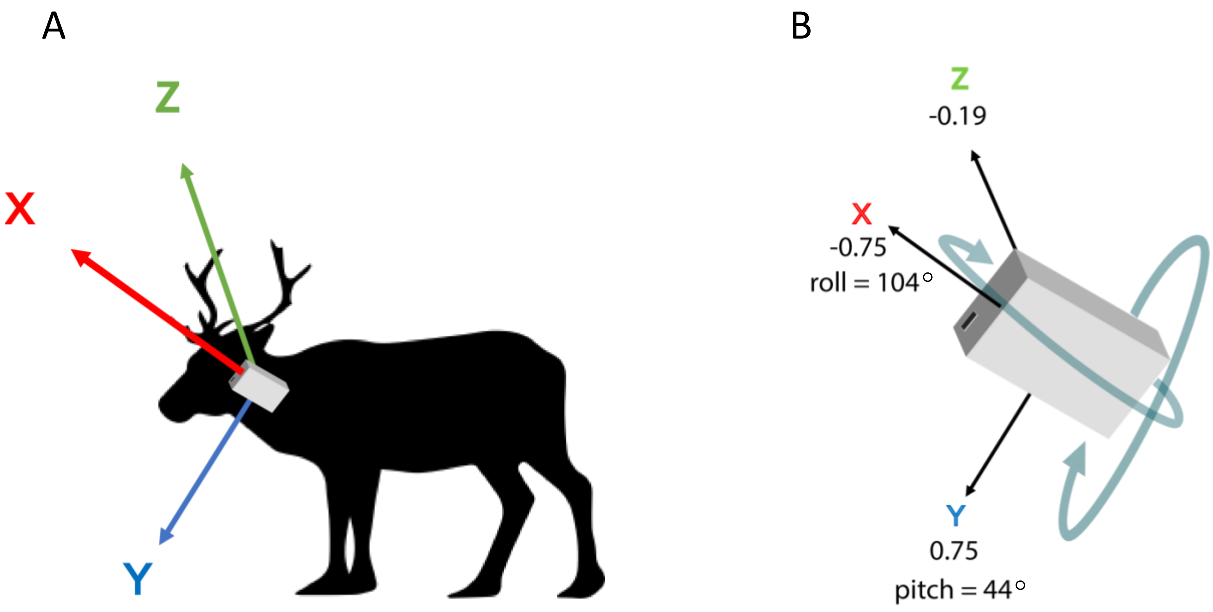

Fig A1. Illustrations of attachment and directions of the accelerometers used on female reindeer (A) to predict seven behavioural classes: 1) grazing, 2) browsing high from a tree, 3) browsing low from a shrub, 4) inactivity, 5) walking, 6) running, and 7) other activities. Mean axis-values (X, Y, Z) and estimated pitch and roll in degrees when the accelerometer is placed on the collar and reindeer is standing still (B).

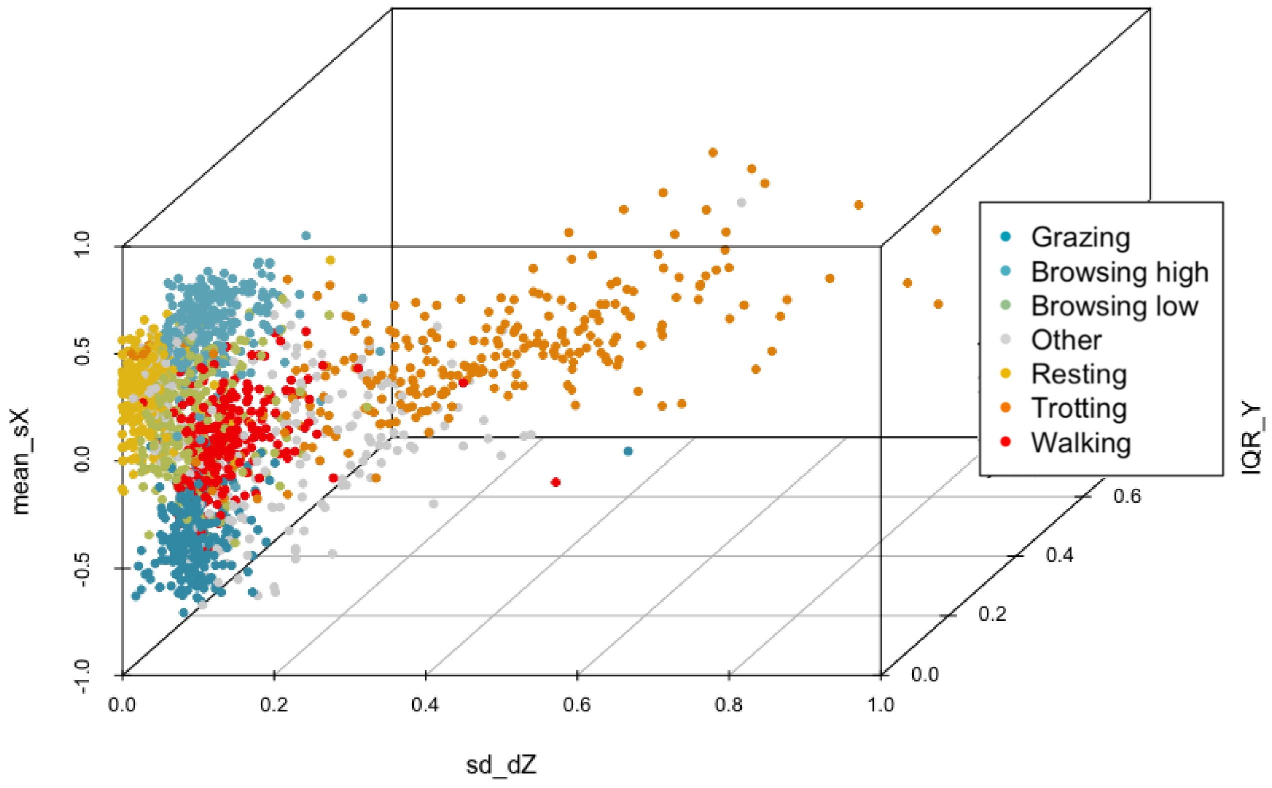

Fig A2. Data distribution using three statistical features 1) mean static acceleration of X-axis (mean_sX), 2) standard deviations of dynamic acceleration of the Z-axis (sd_dZ) and 3) interquartile range of Y-axis (IQR_Y) when using two second windows for segmentation.

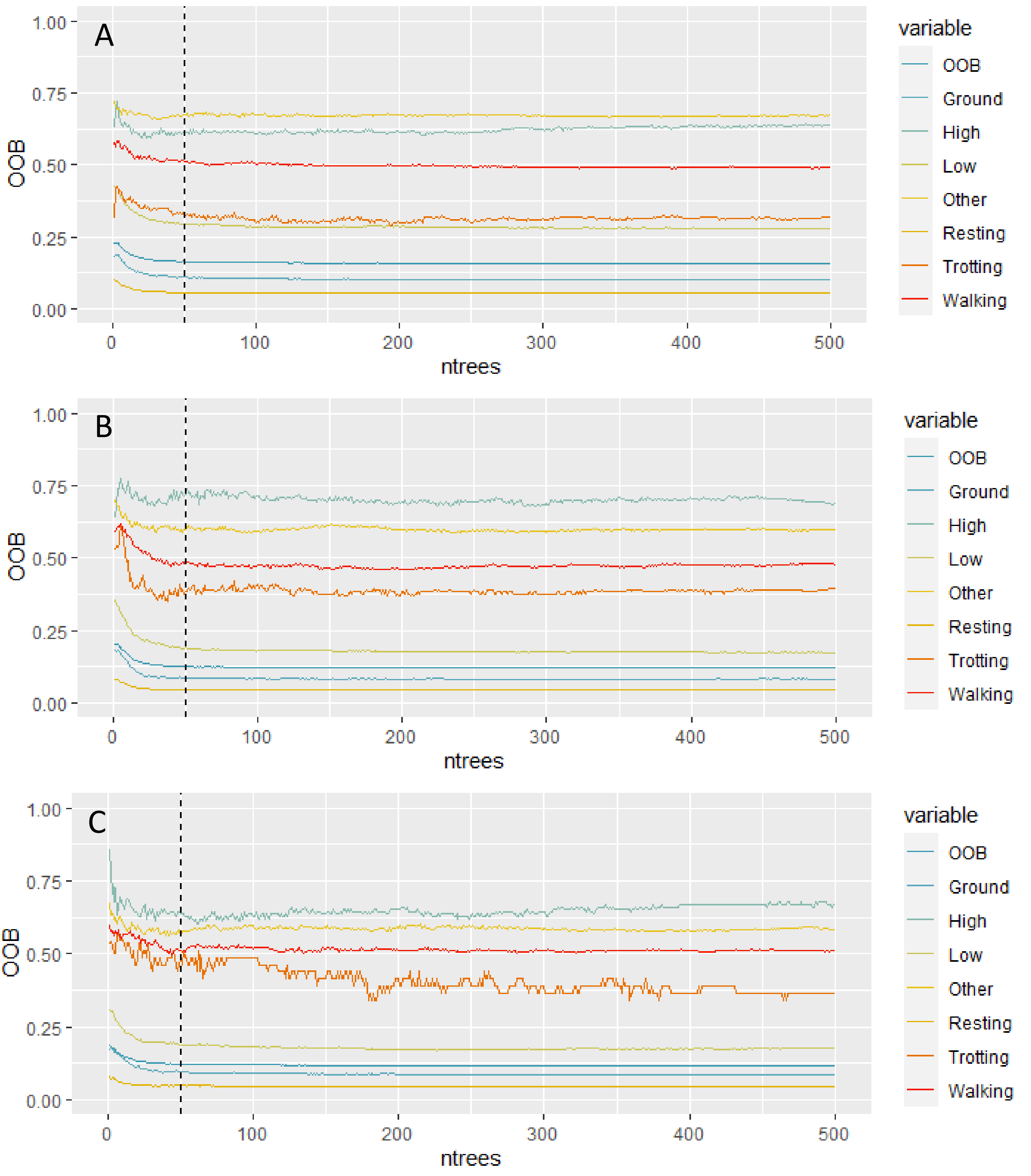

Fig A3. Out-of-bag error (OOB) for seven behavioral classes and overall OBB (black line). Threshold at 50 trees (ntree = 50) was set to maximum trees for random forest (dashed vertical line) for A) 2s windows, B) 3 s windows and C) 5 s windows to avoid overfitting.
